# Dopamine-induced calcium signaling in olfactory bulb astrocytes

**DOI:** 10.1038/s41598-020-57462-4

**Published:** 2020-01-20

**Authors:** Timo Fischer, Paula Scheffler, Christian Lohr

**Affiliations:** University of Hamburg, Division of Neurophysiology, Hamburg, 20146 Germany

**Keywords:** Cellular neuroscience, Glial biology, Olfactory system

## Abstract

It is well established that astrocytes respond to the major neurotransmitters glutamate and GABA with cytosolic calcium rises, whereas less is known about the effect of dopamine on astroglial cells. In the present study, we used confocal calcium imaging in mouse brain slices of the olfactory bulb, a brain region with a large population of dopaminergic neurons, to investigate calcium signaling evoked by dopamine in astrocytes. Our results show that application of dopamine leads to a dose-dependent cytosolic calcium rise in astrocytes (EC_50_ = 76 µM) which is independent of neuronal activity and mainly mediated by PLC/IP_3_-dependent internal calcium release. Antagonists of both D_1_- and D_2_-class dopamine receptors partly reduce the dopaminergic calcium response, indicating that both receptor classes contribute to dopamine-induced calcium transients in olfactory bulb astrocytes.

## Introduction

Dopamine (DA) is one of the most important modulatory neurotransmitters in the mammalian brain. It has several physiological functions in the central nervous system (CNS) including modulation of voluntary movement, reward, sleep regulation, feeding, affect, attention, cognitive function and olfaction^1^. Besides that, dopamine is involved in diverse diseases, such as schizophrenia, Parkinson’s disease and depression. To accomplish all these functions, there are 11 populations of dopaminergic neurons distributed in the CNS^2^. Dopaminergic neurons in the olfactory bulb (OB) are the most numerous among these cell groups^3^ and are subdivided in two subgroups: the short axon (SA) cells and a subpopulation of external tufted (ET) cells^4^. Within the OB, dopaminergic neurons have been reported to be found mainly in the glomerular layer, which is populated by a variety of different periglomerular interneurons. 10–16% of them are supposed to be dopaminergic^5,6^. A common property of the OB dopaminergic neurons is the ability to co-release dopamine and GABA from separate vesicle pools^7–9^. Dopaminergic neurons are the only catecholaminergic neurons located in the OB, hence they could be recognized by the expression of tyrosine hydroxylase (TH), a rate-limiting enzyme in the biosynthesis of dopamine^10,11^. Dopamine acts on dopamine receptors that are metabotropic G protein-coupled receptors containing seven transmembrane domains. Based on coupling to either Gα_s_,_olf_ proteins or Gα_i/o_ proteins to stimulate or inhibit the production of the second messenger cAMP, respectively, dopamine receptors are classified as D_1_-class receptors (including D_1_R and D_5_R) or D_2_-class receptors (including D_2_R, D_3_R and D_4_R)^12–14^.

Although the actions of dopamine on neurons are well described, its influence on astroglia remains poorly understood. There has been evidence for the presence of dopamine receptors leading to cAMP production and subsequent PKA activation as well as NADH modulation and calcium signaling mediated by D_1_-class receptors in cultured astrocytes of different brain regions^15–17^. In addition to the studies above describing dopamine-induced calcium transients in cultured astrocytes, Jennings *et al*. (2017) and Xin *et al*. (2019) could demonstrate that hippocampal and midbrain astrocytes in acute brain slices respond to dopamine with a cytosolic calcium rise. However, the effect of dopamine on astrocytes in other brain regions such as the olfactory bulb remains unknown and has been investigated in the present study.

We intended to elucidate whether olfactory bulb astrocytes respond to dopamine with calcium signaling and to identify the receptor subtypes involved as well as the origin of the intracellular calcium. Our results show that astrocytes respond to bath application of dopamine with calcium release from internal stores by activation of both D_1_-class and D_2_-class receptors.

## Results

### Distribution of olfactory bulb astrocytes and tyrosine hydroxylase-expressing neurons

**Figure 1:**
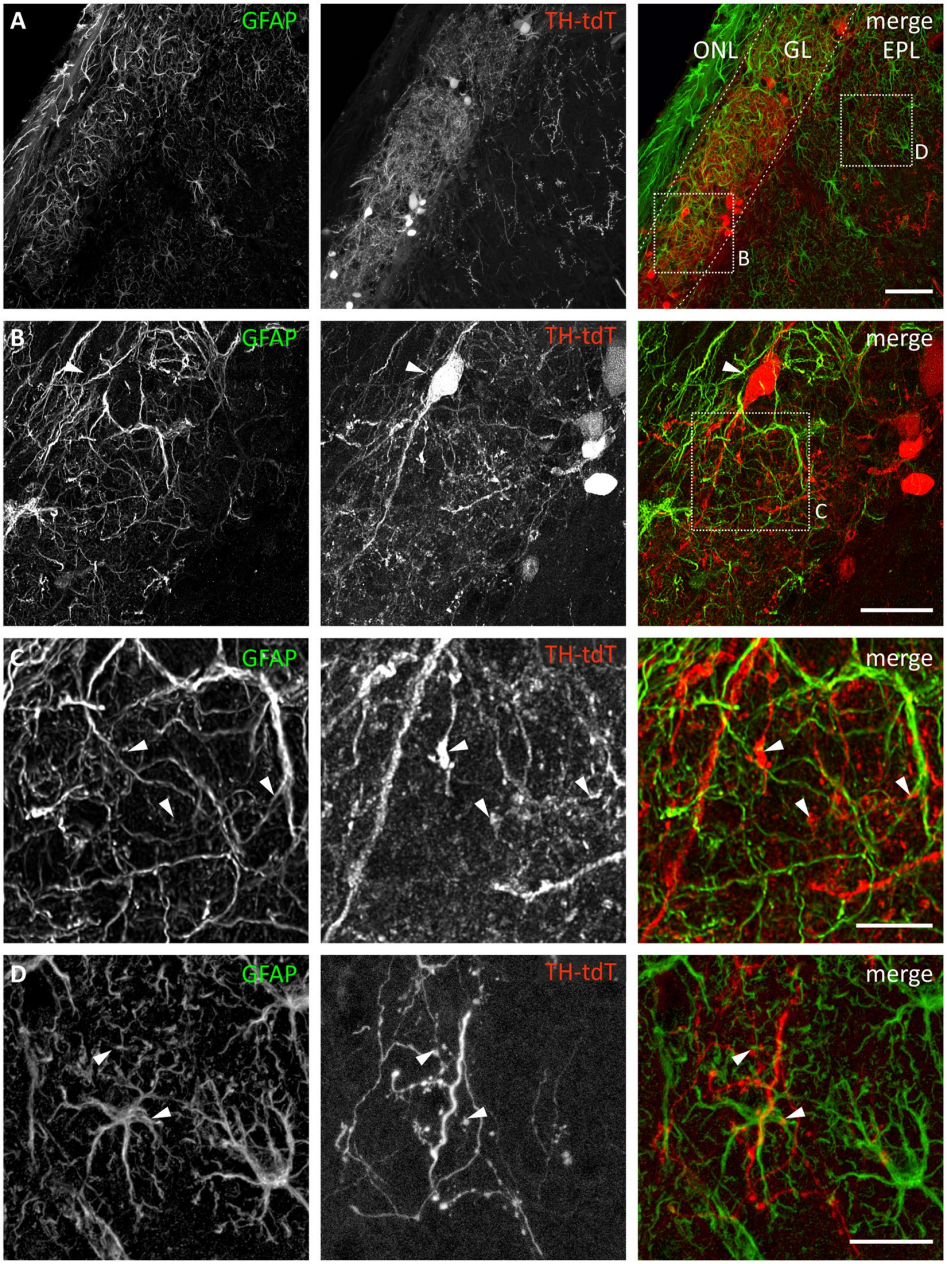
Distribution of astrocytes and tyrosine hydroxylase-expressing neurons in the olfactory bulb. (**A**) GL, glomerular layer; ONL, olfactory nerve layer. Anti-GFAP staining (green) shows astrocytes. Cell bodies of astrocytes are located in the GL and EPL. TH^+^ neurons are labeled by tdTomato in THCre × tdTomato^fl/fl^ mice (red) and are located mainly in the GL, but single neurons expressing tdTomato can be found in the EPL. Scale bar: 50 μm. (**B**) Magnified view from A, highlighting an interglomerular connecting short axon cell (arrowhead) in close proximity to astrocyte processes. Scale bar: 20 µm. (**C**) Magnified view from B, highlighting short axon cell dendrites, varicosities (arrowheads) and astrocytic processes in detail. Scale bar: 10 µm. (**D**) Magnified view from A, highlighting localization of astrocytes and TH^+^ varicosities (arrowheads). Scale bar: 20 µm.

To analyze the distribution of OB astrocytes and TH-expressing neurons in the GL and EPL by immunohistochemistry, we used TH-Cre × tdTomato^fl/fl^ mice and aimed to visualize astrocytes by anti-GFAP staining. GFAP-positive astrocytes were located throughout the GL, which can be distinguished from the ONL by glomerular structures, and the EPL, in which GFAP-expression was weaker compared to the GL (Fig. 1A). TH expression also exhibits its highest density in the GL, but the EPL also contains some TH-positive neurons and neuronal processes (Fig. 1A). In the glomerular layer, dendrites of tdTomato-positive SA cells are closely intermingled with astrocyte processes, suggesting a possible physiological interaction such as neurotransmitter release by SA cells and subsequently activation of astrocytic dopamine receptors (Fig. 1B,C). Close proximity of dendrites of TH-positive neurons and astrocyte processes could also be observed in the EPL, albeit at a lower density (Fig. 1D). In both layers, astrocyte processes were in direct vicinity of tdTomato-positive varicosities, presumptive dopamine release sites^18^.

### Dopamine induces dose-dependent calcium transients in olfactory bulb astrocytes

**Figure 2:**
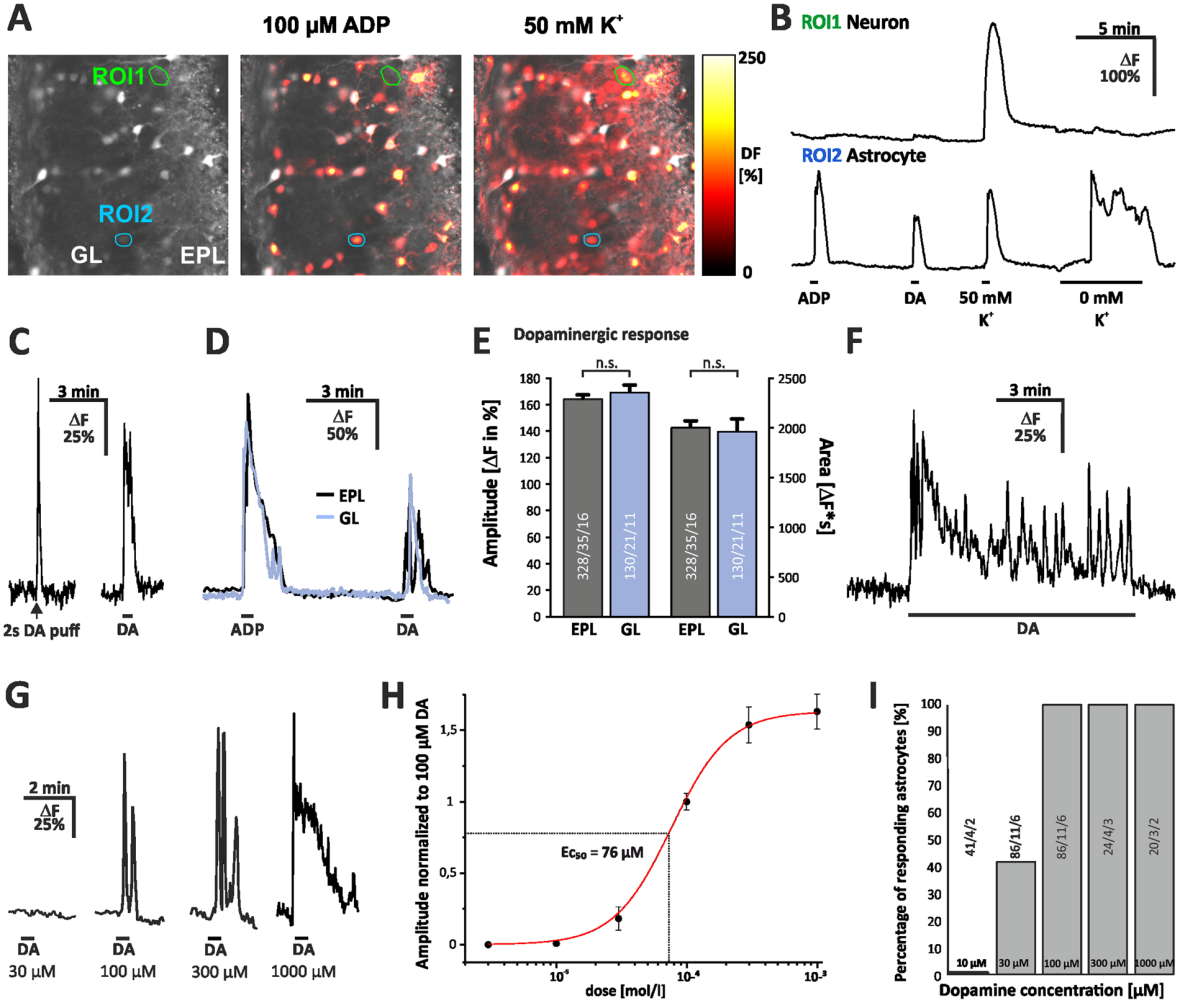
Dopamine-triggered calcium response in olfactory bulb (OB) astrocytes. (**A**) Fluo-8 staining of acute OB slices with example ROIs shown in idle state (left), in the presence of 100 µM ADP (middle) and high potassium ACSF (right). **(B**) Example traces of cells that are considered to be a neuron (ROI1) and an astrocyte (ROI2). **(C**) Dopamine-induced calcium responses of astrocytes by short pressure application (500 µM, left) and bath application (100 µM, right). **(D**) Calcium responses of astrocytes evoked by application of ADP (30 s, 100 µM) and dopamine (DA; 30 s, 100 µM) in both EPL (black trace) and GL (blue trace). (**E**) Averaged amplitudes (left bars) and area (right bars) of DA-induced calcium responses in EPL and GL (error bars: SEM). Sample size as specified in the bars: cells/slices/animals. n.s.: not significant. (**F**) Calcium response of an astrocyte monitored during a 10-min application of DA (100 µM). (**G**) Calcium responses induced by application of dopamine at different concentrations. (**H**) Dose-response curve of dopamine-induced astroglial calcium responses (area), normalized to application of 100 µM DA (+/−SEM). (**I**) Percentage of astrocytes responding to dopamine application.

A general issue in studies about astrocytes using chemical calcium indicators is the identification of astrocytes. Sulforhodamine 101 has been used to identify astrocytes in brain regions such as hippocampus and cortex but fails to label astrocytes in the brain stem^19,20^. We found that astrocytes in the OB also fail to accumulate sulforhodamine 101, however, a well-established approach for OB astrocyte identification is based on their response to ATP, ADP and low concentrations of potassium^21,22^. Removal of external potassium induces calcium influx into astrocytes, but not into neurons (Fig. 2A,B). Hence, the cells that did not respond to K^+^-free saline were presumed to be neurons. These cells also responded to 50 mM K^+^ with a large calcium transient, while 100 µM ADP had no effect on the calcium concentration. Astrocytes, in contrast, not only responded to K^+^-free saline with a calcium increase, but also to application of ADP due to expression of P2Y_1_ receptors^21^ (Fig. 2A,B). According to the results mentioned above, we subsequently used the application of ADP for the identification of astrocytes and to test their viability.

In the present study, we aimed to establish whether olfactory bulb astrocytes *in situ* respond to dopamine application. Results were collected in the glomerular layer and the external plexiform layer that comprise most of the synapses involved in odor information processing in the olfactory bulb^23^. Experiments were performed in the presence of TTX to suppress action potential firing and, hence, neuronal effects on astrocytic calcium. Both short pressure application (500 µM dopamine, 2 s) and bath application (100 µM dopamine, 30 s) led to transient elevation in cytosolic calcium in OB astrocytes (Fig. 2C). Pressure application is a more local stimulation and enables shorter application, followed by short elevations in cytosolic calcium. However, this procedure has considerable disadvantages with regard to the comparability of the experiments. It is not possible to adjust a constant concentration, as the dopamine-containing solution applied to the cells mixes with the bath solution with increasing distance. For this reason we decided to perform all subsequent experiments using bath application. All cells that responded to dopamine with calcium transients did also respond to ADP, which was used to identify astrocytes (see above). Hence, all cells responding to dopamine were identified as astrocytes, while Fluo-8-loaded neurons did not respond to bath application of dopamine with calcium signals (Fig. 2A,B).

As shown in Fig. 2D, bath application of 100 µM dopamine led to transient monophasic or oscillating elevations in cytosolic calcium in both layers. Dopamine application for 30 seconds evoked a calcium response with an amplitude of 163.9+/− 3.5% ΔF and an area of 2000.6+/− 69.1 ΔF*s (n = 328) in the external plexiform layer and 169.1+/− 5.8% ΔF and an area of 1957.4+/− 137.7 ΔF*s (n = 130) in the glomerular layer (Fig. 2E). No significant differences in amplitude and area of the responses were observed between the glomerular layer and the external plexiform layer. Hence, in all following experiments, data of both layers were pooled. Application of 100 µM dopamine for several minutes evoked long-lasting calcium oscillations (Fig. 2F). For the following experiments, we opted for a relatively short application of 30 s. To identify the optimal concentration of dopamine, we established a dose-response curve for bath application of dopamine (Fig. 2G–I), starting with the lowest concentration of 3 µM dopamine and increasing stepwise up to 1000 µM. We monitored the amount of responding astrocytes for every concentration. It became apparent that using 3 µM dopamine (n = 9) none of the astrocytes showed any change in intracellular calcium, while at 10 µM dopamine (n = 41) one of 41 cells displayed a small calcium response. When using 30 µM dopamine (n = 86), 41.6% of the astrocytes showed a response. At 100 µM dopamine (n = 86) and above, every monitored astrocyte generated a prominent calcium elevation, indicating that 100 µM dopamine is sufficient to activate all astrocytes containing dopamine receptors. Furthermore, using a concentration of 100 µM is consistent with the experimentally determined EC_50_ of 76 µM dopamine (Fig. 2H).

### Dopamine-induced calcium transients are independent of neuronal influence and mediated by internal calcium stores

**Figure 3:**
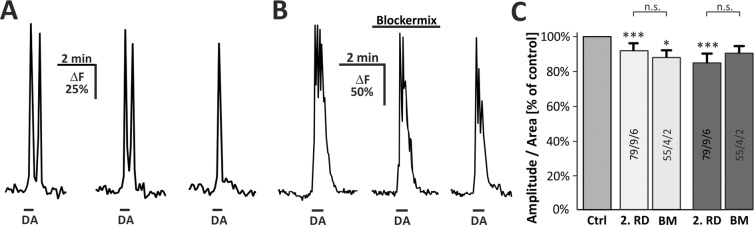
Dopamine-induced calcium transients in OB astrocytes in synaptic isolation. (**A**) Example of multiple applications of DA (100 µM) with a 10-min interval. (**B**) Calcium transients were not affected in presence of GABAergic, glutamatergic und purinergic antagonists (Blockermix contains: NBQX 10 µM, D-APV 50 µM, gabazine 10 µM, CGP55845 10 µM, MPEP 2 µM, MRS2179 50 µM, ZM241385 0.5 µM, TTX 1 µM). (**C**) Normalized averaged amplitudes (light grey, +/−SEM) and area (dark grey, +/−SEM) of calcium responses under control conditions and after application of Blockermix (BM). Results of BM are additionally compared to rundown (RD) experiment as depicted in (**A**) (error bars: SEM). *P < 0.05, **P < 0.01, ***P < 0.005.

The former experiments were performed in the presence of TTX to suppress action potential firing and hence indirect effects by neurons. However, it cannot be excluded that dopamine elicited action potential-independent local calcium rises in neurons that could lead to release of neurotransmitters such as glutamate and GABA. Since glutamate and GABA have been shown to trigger calcium signals in olfactory bulb astrocytes^22,24^, indirect neuronal effects might contribute to the calcium transients in astrocytes evoked by dopamine application. To elucidate whether dopamine-evoked neurotransmitter release contributed to the calcium transients in astrocytes, we investigated dopamine-induced calcium transients in synaptic isolation. We first tested whether repetitive application of dopamine results in rundown of the calcium response, e.g. due to receptor desensitization. After multiple applications of dopamine, a rundown of the dopamine-induced calcium transients could be observed (Fig. 3A). Compared to the first application, the second application evoked calcium transients that were reduced by 8.0+/− 4.6% (n = 79; p < 0.001), whereas calcium transients evoked by the third application were attenuated by 21.6+/− 3.4% (n = 79; p < 0.001; Fig. 3A,C). We then compared responses evoked by dopamine application either in the presence of TTX (1 µM; Suppl. Fig. 1) or in a mix of GABAergic, glutamatergic and purinergic antagonists (GABA_A_: Gabazine 10 µM; GABA_B_: CGP55845 10 µM; AMPA receptors: NBQX 10 µM; NMDA receptors: D-APV 50 µM; mGluR_5_: MPEP 2 µM; P2Y_1_: MRS2179 50 µM; A_2A_: ZM241385 0.5 µM; sodium channels: TTX 1 µM) with the corresponding control application in the absence of receptor antagonists to assess the contribution of these receptors to the dopamine-evoked response (blockermix, BM; Fig. 3B,C). In the presence of the blocker mix, the dopamine-induced calcium transient was slightly decreases by 12.1+/− 3.6% (n = 55; p < 0.05) in amplitude and 9.7+/− 7.4% (n = 55; p < 0.05) in area as compared to the second application of the control experiment (rundown). No significant differences between the values in the control experiments and in experiments using the blockermix were found, indicating that the neuronal contribution to dopamine-evoked calcium transients in OB astrocytes is negligible (Fig. 3C).

**Figure 4:**
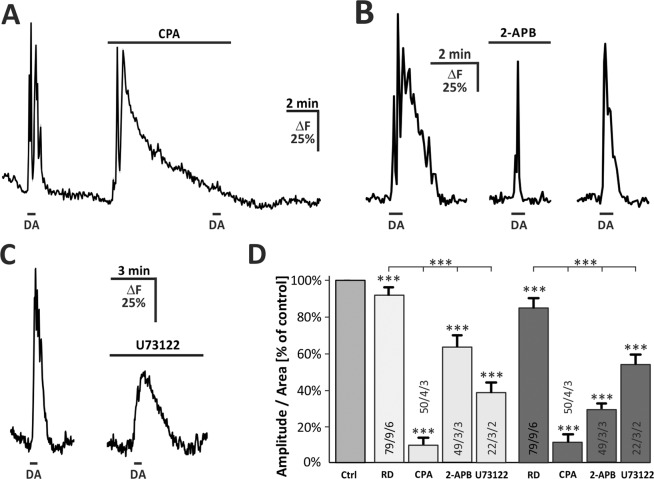
Internal calcium release mediates dopamine-induced calcium transients in OB astrocytes. (**A**) Dopamine-induced calcium transients are entirely suppressed in the presence of SERCA inhibitor cyclopiazonic acid (CPA, 20 µM). (**B**) Calcium transients are partly diminished in the presence of IP_3_ receptor antagonist 2-APB (50 µM). (**C**) Attenuated calcium transients in the presence of PLC inhibitor U73122 (50 µM). (**D**) Normalized averaged amplitudes (left, +/−SEM) and area (right, +/−SEM) of calcium responses under control conditions and after application of CPA, 2-APB and U73122. Results are additionally compared to rundown (RD) experiment as depicted in Fig. 3A. *P < 0.05, **P < 0.01, ***P < 0.005.

To test whether dopamine-evoked calcium transients are mediated by calcium release from internal stores, we applied dopamine before and after calcium stores were depleted by incubation with 20 μM cyclopiazonic acid (CPA). Depletion of internal stores led to a long-lasting elevation in intracellular calcium and suppressed dopamine-evoked calcium responses. The mean amplitude of dopamine-induced calcium transient was reduced by 93.8+/− 2.5% (n = 50; p < 0.001) and the area by 88.2+/− 2.9% (n = 50; p < 0.001) (Fig. 4A,D), indicating that the calcium rise is mainly mediated by internal calcium release. The canonical pathways downstream of dopamine receptors are stimulation or inhibition of adenylate cyclase via Gα_s_ and Gα_i_ proteins, however, intracellular signaling via Gα_q/11_ proteins and phospholipase C (PLC) has also been described by both D_1_-class and D_2_-class dopamine receptors^25–29^. To ascertain whether the calcium rise depends on the PLC/IP_3_ signaling pathway, we applied dopamine in the presence of the IP_3_ receptor antagonist 2-APB (Fig. 4B). A relatively low concentration of 50 µM 2-APB was used, since higher concentrations activate transient receptor potential channels TRPV and lead to calcium oscillations in OB astrocytes^24^. 50 µM 2-APB attenuated dopamine-induced calcium responses by 36.8+/− 6.6% (n = 49; p < 0.001) in amplitude and 71.3+/− 3.1% (n = 49; p < 0.001) in area, suggesting the involvement of IP_3_ receptors (Fig. 4B,D). In addition, the sustained phase of the calcium response was entirely blocked by 2-APB (Fig. 4B). In order to obtain further indication for the participation of this signaling pathway, dopamine was applied after 30 minutes of incubation with the PLC inhibitor U73122 (50 µM). Under these conditions, the dopamine-induced calcium response was reduced by 61.6+/− 5.2% (n = 22; p < 0.001) in amplitude and by 46.2+/− 5.0% (n = 22; p < 0.001) in area (Fig. 4C,D). Interestingly, the dopamine-evoked calcium transient appeared to be sustained in the presence of U73122 as compared to the control response (Fig. 4C).

### Dopamine-induced calcium transients depend on D1 and D2-like receptors

**Figure 5:**
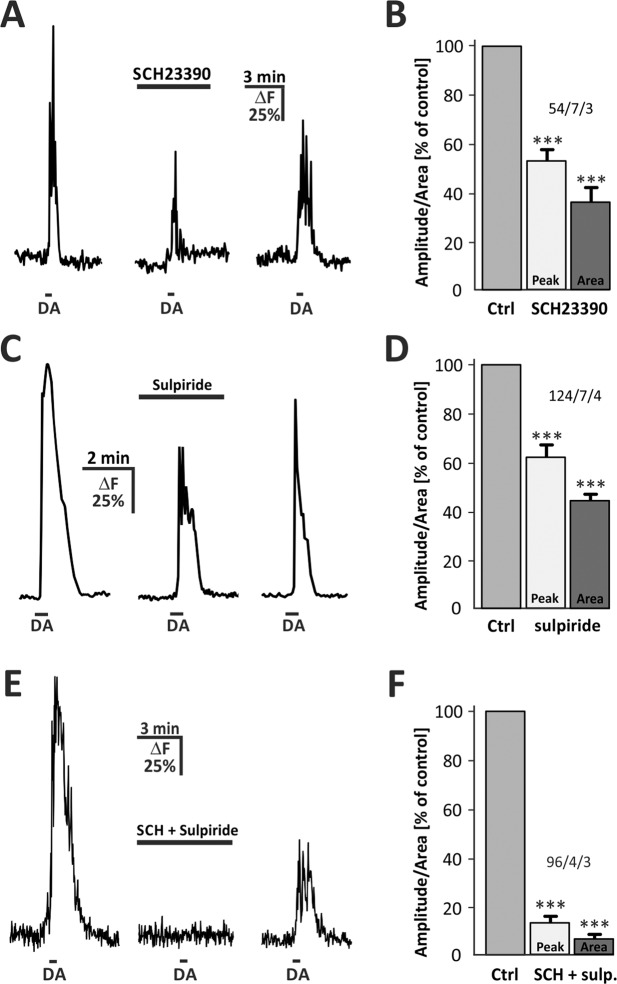
Effect of DA receptor antagonists on dopamine-induced calcium signaling in OB astrocytes. (**A**) Calcium transients evoked by DA (30 s, 100 µM) where reduced in amplitude and integral in the presence of D1-class antagonist SCH23390 (50 µM). (**B**) Normalized averaged amplitude and area of calcium responses in presence of SCH23390. (**C**) Calcium transients where reduced in the presence of D2-class antagonist sulpiride (50 µM). (**D**) Normalized averaged amplitude and area of calcium responses in presence of sulpiride. (**E**) Effect of both SCH23390 and sulpiride on dopamine-evoked calcium responses. (**F**) Normalized averaged amplitude and area (+/−SEM) of calcium responses in the presence of both antagonists. *P < 0.05, **P < 0.01, ***P < 0.005.

To accomplish the characteristics of astrocytic dopamine signaling, we were interested which class of dopamine receptor is expressed and mediates calcium signaling in OB astrocytes. Therefore, we tested the effects of D_1_-class as well as D_2_-class dopaminergic receptor antagonists on dopamine-induced calcium transients. In the presence of the D_1_-antagonist SCH23390, the dopamine-induced calcium transient was reduced by 47.2+/− 5.0% (n = 54; p < 0.001) in amplitude and 64.1+/− 6.2% (n = 54; p < 0.001) in area (Fig. 5A,B). The D_2_-antagonist sulpiride resulted in an attenuation of the dopamine-induced calcium transient by 37.9+/− 4.1% (n = 124; p < 0.001) in amplitude and 48.2+/− 6.9% (n = 124; p < 0.001) in area (Fig. 5C,D). In the presence of both SCH23390 and sulpiride, dopamine-dependent calcium signaling was nearly completely abolished with a reduction by 85.9+/− 2.8% (n = 96; p < 0.001) in amplitude and 93.1+/− 1.7% (n = 96; p < 0.001) in area, respectively (Fig. 5E,F). In all experiments, the antagonist-dependent decrease in calcium rise was at least partly reversible. These results indicate a participation of both D_1_-class and D_2_-class receptors in dopamine-induced calcium signaling in OB astrocytes.

## Discussion

### Dopamine induces calcium transients in olfactory bulb astrocytes

In the present study we showed that dopamine induced an increase in intracellular calcium concentration in olfactory bulb astrocytes *in situ*. We could demonstrate that the dopamine-evoked calcium rise was entirely blocked in the presence of SERCA inhibitor CPA, indicating that dopamine triggered calcium release from internal stores such as the endoplasmic reticulum (ER). The most common mechanism to liberate calcium from the ER is the PLC/IP_3_ signaling pathway, which has previously been shown in OB astrocytes to be activated by P2Y_1_ receptors, A_2A_ receptors and mGluR_5_^21,30^. Although dopamine receptors are mainly considered to be linked to adenylate cyclases, several instances have shown stimulation of PLC by both D_1_-class and D_2_-class dopamine receptors (reviewed by Beaulieu *et al*.^14^). Hence, it is likely that dopamine releases calcium from the ER by stimulation of PLC. Based on this assumption, we performed experiments in the presence of phospholipase C inhibitor U73122 and the IP_3_ receptor antagonist 2-APB, which both resulted in a considerable decrease in dopamine-induced calcium rise. Furthermore, the kinetics of the calcium response was altered in the presence of both 2-APB and U73122. The property of 2-APB acting not only as an IP_3_ receptor antagonist, but also as an inhibitor of store-operated calcium entry (SOCE), may provide an explanation for the rapid decay of the calcium rise after reaching its peak, since inhibition of SOCE channels suppresses the late phase of the calcium response^31^. This result may be considered as a first indication of the involvement of SOCE in dopamine-induced calcium transients in astrocytes. In contrast, in the presence of the PLC inhibitor U73122, despite a significant reduction in mean amplitude, a prolongation of the dopamine-induced calcium transient could be observed. This might be due to the U73122-mediated attenuation of the PKC signaling pathway, which acts downstream of PLC activation as a negative feedback mechanism to terminate calcium elevations evoked by G protein-coupled receptors^32,33^. Our results suggest major contribution of the PLC/IP_3_ pathway to dopaminergic calcium responses in OB astrocytes, however, both U73122 and 2-APB failed to entirely block dopamine-induced calcium transients. We cannot exclude that the concentrations of the compounds at their targets were not sufficient to totally block PLC-mediated IP_3_ receptor activation, since U73122 does not easily diffuse within tissue and, in order to avoid activation of TRPV channels, the concentration of 2-APB could not be raised to values needed for complete block of IP_3_ receptors^24^.

### D_1_- and D_2_-class receptors mediate calcium signaling in olfactory bulb astrocytes

The dopamine-induced calcium elevation was decreased by both D_1_- and D_2_-class receptor antagonists, thus both classes of dopamine receptors appear to contribute to the dopamine-evoked calcium transients. This is in line with observations suggesting that both D_1_- and D_2_-class receptors elicit release of IP_3_ and calcium signaling in astrocytes^25–29^. Whether this effect in OB astrocytes is mediated by D_1_-D_2_ heteromers, as shown in striatal neurons^34^, or by independent D_1_ and D_2_ receptors is not known and needs further investigation. In contrary to our results, studies in the hippocampus and ventral midbrain have shown that activation of D_2_ receptors leads to a decrease in cytosolic calcium, whereas D_1_ receptors mediate a calcium rise^29,35^. The D_2_-class receptor-mediated decrease in calcium in hippocampal astrocytes presumably results from modulation of L-type voltage-gated calcium channels^29^, which has also been shown in nucleus accumbens neurons^36^. Apparently, this mechanism does not exist in astrocytes of the olfactory bulb. However, it must me mentioned that, due to the limitations of two-dimensional confocal imaging and lack of visibility of small cell processes after bulk loading of calcium indicators, it is possible that other small-scaled but functionally important calcium signals in fine astrocyte processes may have been unexploited. Furthermore, in comparison to the work of Jennings *et al*.^29^, there are some considerable differences in experimental design, such as our method of application differed in terms of duration. While Jennings *et al*. used a puff application of 3 minutes and bath application of 10 minutes in most trials, it was sufficient to confine the dopamine application to 2 seconds (puff application) and 30 seconds (bath application) in our study, suggesting a high dopamine sensitivity in astrocytes of the OB, but only moderate sensitivity in the hippocampus. This is confirmed by the long delay in onset of the calcium response often seen in hippocampal astrocytes^29^. The dopamine sensitivity of OB astrocytes might even be underestimated, since bulk-loading of calcium indicators results in significantly smaller calcium-dependent fluorescence changes compared to loading single cells (and gap junction-coupled cells) via a patch pipette^29^.

Although the localization of tyrosine hydroxylase and thus dopamine-containing neuronal elements has its highest density in the glomerular layer (Fig. 1A)^10^, the distribution of the D_1_-receptor is widely spread over all OB layers except of the nerve layer^37–42^. This indicates a functional role of dopamine in the entire OB, including the glomerular layer as well as in external plexiform layer, which is consistent with our results. The distribution of the D_2_ receptor has its highest density in the GL and nerve layer, while D_2_ receptor expression in the EPL is sparse^40,43–45^. Despite that, the dopamine-induced calcium transients where strongly reduced in the presence of D_2_ antagonist sulpiride in both the GL and EPL, indicating expression of D_2_ receptors in astrocytes of the EPL. Previous studies by Pignatelli *et al*.^37^ showed that during the process of maturation of adult-born dopaminergic neurons, two populations of TH^+^ neurons outside the glomerular layer exist. One of these populations represents dopaminergic neurons that undergo the last step in maturation and can be found in the mitral cell layer and external plexiform layer^37^. These cells project numerous processes within the EPL, suggesting release of dopamine not only in the GL but also in deeper layers. As shown in Fig. 1, our histological analysis of TH-expressing neurons supports this observation. Hence, astrocytes in the EPL might detect dopamine released by immature adult-born neurons with calcium signaling and, in turn, provide growth factors and components of the extracellular matrix to support neuronal maturation as shown, e.g., in the cerebellum^46^.

### Putative functional roles of dopamine-evoked calcium signaling in astrocytes

It is well known that astrocytes are involved in a variety of processes in the CNS. Apart from well-established supportive astrocytic functions such as metabolic supply and potassium homeostasis, more recent studies revealed that astrocytes are tightly integrated into neural networks in a functional manner. As a result of astroglial calcium excitability, astrocytes are capable of “gliotransmission”, i.e. release of transmitters which can lead to signaling to neighboring neurons and blood vessels^47,48^. In the main olfactory bulb, several studies provided evidence for important functions of astrocytes, e.g. gliotransmission^49^. Kozlov *et al*. demonstrated that astrocytes in the rat olfactory bulb release two classical transmitters, GABA and glutamate, upon mechanical stimulation to evoke synchronous currents in mitral and granule cells^50^. Another gliotransmitter released by OB astrocytes is ATP that is degraded to adenosine, a potent neuromodulator in the OB^23,51^. In mitral cells, adenosine activates two-pore domain potassium channels and inhibits presynaptic calcium channels, resulting in reduced spontaneous firing and attenuated reciprocal dendro-dendritic inhibition^52,53^. OB astrocytes also play a key role in neurovascular coupling, since it has been shown that the physiological activation of olfactory sensory neurons by odors reliably triggers calcium increases in astrocyte processes in the glomerular layer and subsequent dilation of blood vessels^30,54^. Neurovascular coupling is not restricted to the GL, since astrocytes transmit panglial calcium waves to olfactory ensheathing glial cells in the nerve layer, which there contribute to regulation of blood vessel diameter^55,56^. Which role dopaminergic calcium signaling in astrocytes plays in neuronal performance and neurovascular coupling in the OB is not known so far and needs further investigation. However, tuning of olfactory information processing and odor perception by neuromodulators such as dopamine, noradrenaline, acetylcholine and serotonin has moved into the focus of research on olfaction, in particular in recent years^23,57–59^, and it is becoming increasingly clear that astrocytes have to be considered to take actively part in these processes. This hypothesis appears to be supported by findings showing interactions of dopamine and astrocytes in other regions of the CNS, such as ventral midbrain and hippocampus^29,35^.

## Methods

### Animals used for slice preparation

Naval Medical Research Institute (NMRI) outbred mice from postnatal day 7 (p7) to p21 were used for calcium imaging experiments. The mice were bred in the institute’s animal facility at the University of Hamburg. This study was carried out in accordance with the recommendations of the European Union’s and local animal welfare guidelines. Mice were anesthetized using isoflurane (5% mixed with 1 L/min O_2_) and decapitated before using for experiments. The permission to despatch mice for the purpose of organ extraction was obtained by the Hamburg Authority of Health and Consumer Protection (GZ G21305/591-00.33; Behörde für Gesundheit und Verbraucherschutz, Hamburg, Germany). According to the local laws, an additional ethical approval for animal experiments in the current study was not required. Olfactory bulb slices were prepared as described before^60^. Slices were quickly transferred into a chilled artificial cerebrospinal fluid (ACSF, see below). 200 µm thick horizontal slices of the bulbs were cut using a vibratome (Leica VT1200S, Bensheim, Germany). Brain slices were stored in ACSF for 30 min at 30 °C and 15 min at room temperature before starting experiments. ACSF was continuously gassed with carbogen (95% O2/5% CO2; buffered to pH 7.4 with CO2/bicarbonate).

### Solutions and chemicals

The standard ACSF for acute brain slices contained (in mM): NaCl 125, KCl 2.5, CaCl_2_ 2, MgCl_2_ 1, D-glucose 25, NaHCO_3_ 26, NaHPO_4_ 1.25, gassed during the entire experiment with carbogen to adjust the pH to 7.4. MRS 2179 (2′-deoxy-N6-methyladenosine 3′, 5′-bisphosphate tetrasodium salt) and ZM241385 (4-(2-[7-Amino-2-(2-furyl)[1, 2, 4]triazolo[2, 3-a][1, 3, 5]triazin-5-ylamino]ethyl)phenol) was obtained from Tocris (Bristol, UK). D-APV (D-2-amino-5-phosphonopentanoic acid), NBQX (2, 3-dioxo-6-nitro-1, 2, 3, 4-tetrahydrobenzo[f]quinoxaline-7-sulfonamide), gabazine (6-Imino-3-(4-methoxyphenyl)-1(6 *H*)-pyridazinebutanoic acid hydrobromide), TTX (tetrodotoxin, Octahydro-12-(hydroxymethyl)-2-imino-5, 9:7, 10a-dimethano-10a*H*-[1, 3]dioxocino[6, 5-*d*] pyrimidine-4, 7, 10, 11, 12-pentol + citrate buffer), CPA (cyclopiazonic acid; (6a*R*, 11a*S*, 11b*R*)-10-acetyl-2, 6, 6a, 7, 11a, 11b-hexahydro-11-hydroxy-7, 7-dimethyl-9*H*-pyrrolo[1′, 2′:2, 3]isoindolo[4, 5, 6-*cd*]indol-9-one, SCH23390 ((R)-(+)-7-chloro-8-hydroxy-3-methyl-1-phenyl-2, 3, 4, 5-tetrahydro-1H-3-benzazepine) and sulpiride ((*R*, *S*)-(±)-5-Aminosulfonyl-*N*-[(1-ethyl-2-pyrrolidinyl)methyl]-2-methoxybenzamide) were obtained from Abcam (Cambridge, United Kingdom). All substances were stored as stock solutions according to the manufacturers’ description.

### Immunohistochemistry

Immunohistochemistry on olfactory bulbs of TH-Cre (B6.Cg-*7630403G23Rik*^*Tg(Th-cre)1Tmd*^/J) × tdTomato^fl/fl^ mice (P28) was performed as described^22,61,62^. These mice express the red fluorescent reporter tdTomato under control of Cre recombinase, which is expressed only by cells with active tyrosine hydroxylase promoter. After dissection, the olfactory bulbs were kept for 1 h at room temperature (RT) in formalin (4% paraformaldehyde in PBS, pH 7.4). Subsequently, 120 μm thick sagittal slices were prepared with a vibratome (VT1000S, Leica, Nussloch, Germany) and incubated for 1 h in blocking solution (10% normal goat serum [NGS], 0.5% Triton X-100 in PBS) at RT. Afterwards, the slices were incubated for 48 h at 4 °C with a chicken anti-GFAP antibody (Synaptic Systems, Göttingen, Germany; 1:1000). The antibody was diluted in 1% NGS, 0.05% Triton X-100 in PBS. Slices were incubated in PBS with the goat anti-chicken Alexa 488 secondary antibody (Abcam; 1:1000) for 24 h at 4 °C. Subsequently, the slices were mounted on slides using self-hardening embedding medium (Immu-Mount, Thermo Fisher). Immunohistological stainings were analyzed using a confocal microscope (C1 Eclipse, Nikon, Düsseldorf, Germany). Confocal images were deconvolved using a theoretical point-spread function (Huygens, SVI, Hilversum, Netherlands) and enhanced for brightness and contrast using ImageJ and Adobe Photoshop CS2.

### Calcium imaging

Slices were incubated with the membrane-permeable form of the calcium indicator Fluo-8 (Fluo-8-AM; 2 µM in ACSF) made from a 2 mM stock solution (dissolved in DMSO and 20% pluronic acid) for 30 min. Brain slices were then placed in the recording chamber and fixed with a U-shaped platinum wire with nylon strings. Brain slices were continuously superfused at a rate of 2 ml/min with ACSF that was gassed with carbogen (95 O_2_/5% CO_2_). Bath perfusion with ACSF was accomplished using a peristaltic pump (Vario, Ismatec, Germany). Drugs were applied via the perfusion system. If not stated otherwise, ACSF contained 1 µM TTX to suppress neuronal activity. Changes in intracellular calcium levels in olfactory bulb astrocytes were recorded by confocal microscopy (C1 Eclipse, Nikon, Düsseldorf, Germany). An excitation laser wavelength of 488 nm and a frame rate of 0.75 fps were used and the fluorescence was collected through a 500–530 nm bandpass filter.

### Data analysis and statistic

The data were evaluated with Nikon EZ-C1 Viewer (Nikon) and statistical tests have been applied with Origin Pro 9.1 (OriginLab Corporation, Northampton, USA). To analyze changes of the calcium level in astrocytes, cell somata were marked as regions of interests (ROIs). Cells located in the glomerular layer and external plexiform layer that showed a calcium response to ADP were identified as astrocytes^21,22^. This method of cell identification has been verified by using K^+^-free and 50 mM K^+^-containing ACSF, in which K^+^ was exchanged for Na^+^. Astrocytes, but not neurons, respond to K^+^-free ACSF with calcium signaling^31^. The astrocytic calcium elevations have been observed in both glomerular layer and external plexiform layer, without a significant difference in amplitude or duration, therefore most of the results in the present study are pooled data of both layers. To evaluate changes of calcium levels over time, Fluo-8 fluorescence intensity (F) was recorded during the course of the experiment and normalized to the basal fluorescence intensity in absence of pharmacological stimuli. Changes in calcium are given by ΔF as the percentage changes in fluorescence with respect to the basal fluorescence. Calcium responses were assessed as amplitude (baseline to peak) and area (integral of the curve from beginning of the response until baseline was reached again). All values are given as mean values (+/−) standard error of the mean with “n” representing the number of cells analyzed for a certain set of experiment. In the figures, sample size is given as “number of cells/number of brain slices/number of animals”. The proof of statistical significance was done by Wilcoxon signed-rank test at an error probability p (*p < 0.05; **p < 0.01; ***p < 0.001). In case of independent data the Mann-Whitney-U test was applied. The dose-response curve was generated using the response to 100 µM dopamine, a concentration used in all measurements analyzed for the dose-response relationship, as reference. The values of all responses to 100 µM dopamine were averaged, resulting in 85.2+/− 5.1% ΔF, and ΔF values of all individual cells were normalized to this value. Normalized values were pooled and the dose-response curve was generated using Origin Pro 9.1.

## Supplementary information

Supplementary Information.

Supplementary Information.

## Data Availability

All data are available from the authors upon request.
