# Supplementary figures and images for: Dopamine-induced calcium signaling in olfactory bulb astrocytes
Source: Sci Rep. 2020 Jan 20;10:631. doi: 10.1038/s41598-020-57462-4 (PMC6971274; doi:10.1038/s41598-020-57462-4)

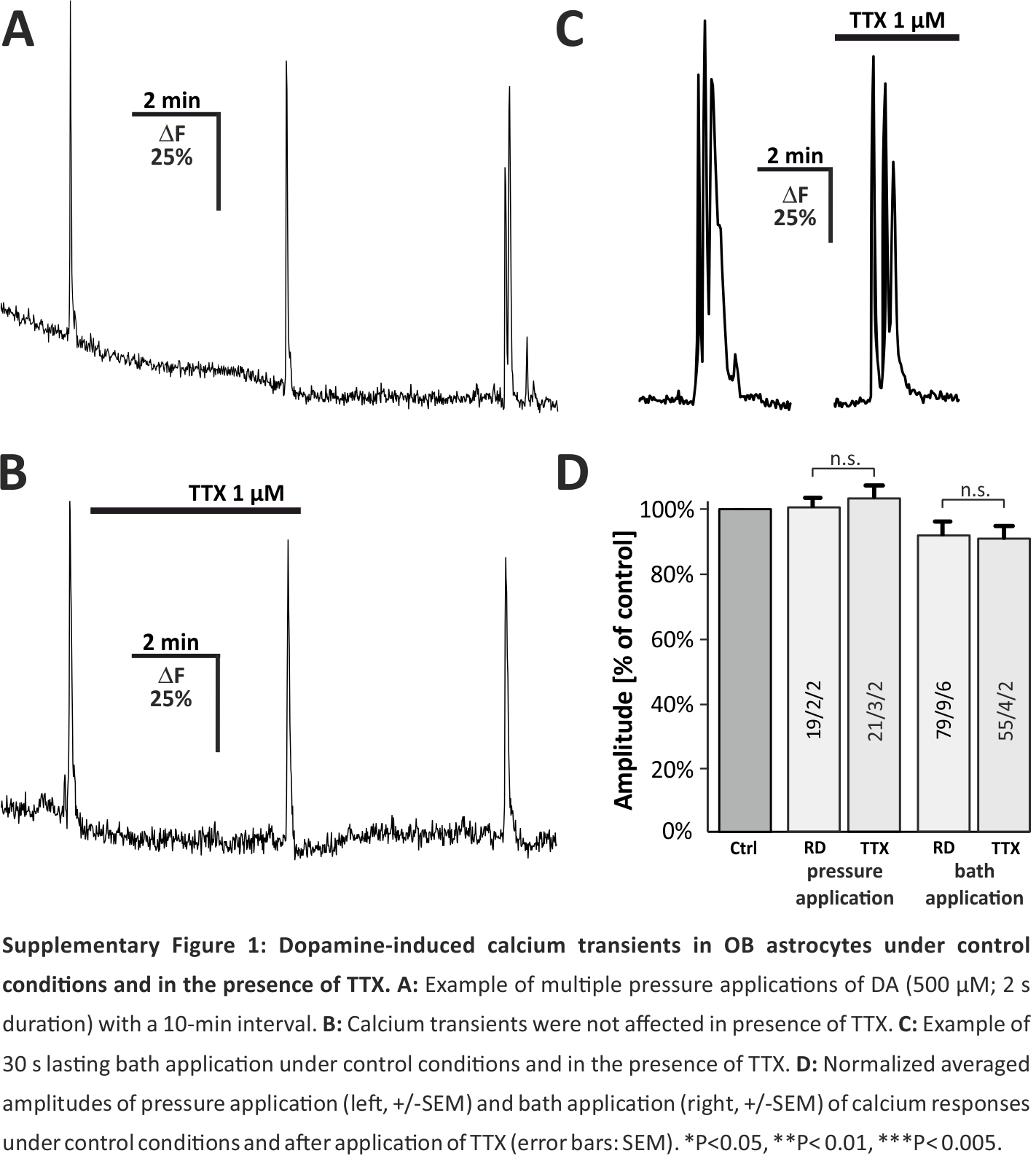

Supplement: Supplementary file 1 — Supplementary Information. [file 41598_2020_57462_MOESM1_ESM.tif]
