# Supplementary material for: Dopamine-induced calcium signaling in olfactory bulb astrocytes
Source: Sci Rep. 2020 Jan 20;10:631. doi: 10.1038/s41598-020-57462-4 (PMC6971274; doi:10.1038/s41598-020-57462-4)
Supplement: Supplementary file 2 — Supplementary Information. [file 41598_2020_57462_MOESM2_ESM.docx]

**Supplementary Figure 1: Dopamine-induced calcium transients in OB astrocytes under control conditions and in the presence of TTX. A:** Example of multiple pressure applications of DA (500 µM; 2 s duration) with a 10-min interval. **B:** Calcium transients were not affected in presence of TTX. **C:** Example of 30 s lasting bath application under control conditions and in the presence of TTX. **D:** Normalized averaged amplitudes of pressure application (left, +/-SEM) and bath application (right, +/-SEM) of calcium responses under control conditions and after application of TTX (error bars: SEM). *P<0.05, **P< 0.01, ***P< 0.005.
